# Distinct Expression Levels of *ALS, LIP*, and *SAP* Genes in *Candida tropicalis* with Diverse Virulent Activities

**DOI:** 10.3389/fmicb.2016.01175

**Published:** 2016-07-29

**Authors:** Shuanbao Yu, Wenge Li, Xiaoshu Liu, Jie Che, Yuan Wu, Jinxing Lu

**Affiliations:** State Key Laboratory of Infectious Disease Prevention and Control, Collaborative Innovation Center for Diagnosis and Treatment of Infectious Diseases, National Institute for Communicable Disease Control and Prevention, Chinese Center for Disease Control and PreventionBeijing, China

**Keywords:** *Candida tropicalis*, candidosis, virulence factor, virulence gene expression, cell damage

## Abstract

*Candia tropicalis* is an increasingly important human pathogen, causing nosocomial fungemia among patients with neutropenia or malignancy. However, limited research has been published concerning its pathogenicity. Based on the phenotypes of *C. tropicalis* in our previous study, we selected nine representative strains with different activities of virulence factors (adhesion, biofilm formation, secreted aspartic proteinases, and hemolysins), and one reference strain, ATCC750. The present study aimed to investigate the filamentation ability, the expression of virulence genes (*ALST1-3, LIP1, LIP4*, and *SAPT1-4*) and the cell damage of *C. tropicalis* strains with diverse virulences. *C. tropicalis* exhibited strain-dependent filamentation ability, which was positively correlated with biofilm formation. Reverse transcriptase PCR analysis showed that the *ALST3* and *SAPT3* genes had the highest expression in their corresponding genes for most *C. tropicalis*. The expressions of virulence genes, except *ALST3* on polystyrene, were upregulated compared with growth in the planktonic and on human urinary bladder epithelial cell line (TCC-SUP) surface. Clustering analysis of virulence genes showed that isolates had a high biofilm forming ability on polystyrene formed a group. Lactate dehydrogenase assays showed that the cell damage induced by *C. tropicalis* markedly increased with longer infection time (24 and 48 h). Strain FXCT01, isolated from blood, caused the most serious cell damage; while ZRCT52, which had no filamentation ability, caused the least cell damage. Correlation analysis demonstrated significant correlation existed between adhesion on epithelial cells or the expression of *ALST2-3* and cell damage. Overall, our results supported the view that adhesion and filamentation may play significant roles in the cell damage caused by *C. tropicalis*.

## Introduction

*Candida tropicalis*, an emerging opportunistic pathogen, mainly causes superficial and invasive infections in human populations, especially among neutropenic patients and those with hematological malignancies (Guinea, 2014). However, less is known about the pathogenicity of *C. tropicalis* compared with the more extensively studied *C. albicans* (Silva et al., 2011b). Several factors were reported to contribute to *Candida* pathogenicity, including adhesion to medical devices and host cells; biofilm formation (BF); filamentation ability; and secretion of hydrolytic enzymes, including secreted aspartyl proteases (Saps), esterases, lipases, phospholipases, and hemolysins (Silva et al., 2011b; Lackey et al., 2013; Hirakawa et al., 2015).

Adherence to host cells is the first step in invasive infections by *Candida* and in the BF, which plays a vital role in its pathogenicity (Ramage et al., 2006; Silva-Dias et al., 2015). Filamentation is also required for virulence-related processes, including invasion of epithelial cell layers, and BF (Jayatilake et al., 2006; Lackey et al., 2013). PCR using consensus primers identified at least three Agglutinin-like sequences (*ALST1-3*) for adhesion of *C. tropicalis* (Hoyer et al., 2001). In addition, Buter et al. used a phylogenomic approach to identify 16 genes in *ALS* gene family in *C. tropicalis* (Butler et al., 2009). In addition, hydrolytic enzymes might also play an important role in infection by disrupting host mucosal membranes, degrading immunological and structural defense proteins, and providing nutrients (Buzzini and Martini, 2002; Silva et al., 2012; Rossoni et al., 2013). *C. tropicalis* possesses four Saps-encoding genes (*SAPT1-4*), and five secreted lipase-encoding genes (Togni et al., 1991; Zaugg et al., 2001; Butler et al., 2009). The specific gene sequences of *SAPT1-4, LIP1*, and *LIP4* could be found in GenBank.

The virulence phenotypes (adhesion, BF, Saps, phospholipases, and hemolysins) of 68 *C. tropicalis* were analyzed *in vitro* in our previous study (Yu et al., 2015). However, limited studies have been performed on virulence factors of *C. tropicalis* at the level of transcription. According to the activities of virulence factors in *C. tropicalis*, we selected nine representative strains and one reference strain, ATCC750, in this study. The objective of this study was to determine the activities of the virulence factors (lipase, esterase, and filamentation ability) *in vitro*; to explore the expression profile of virulence genes, including *ALST1-3, LIP1, LIP4*, and *SAPT1-4* in planktonic, on polystyrene and human urinary bladder epithelial cell line (TCC-SUP; ATCC HTB-5™) surfaces; to examine correlation between virulence gene expression and the phenotype; and to analyze the specific roles of virulence factors in cell damage phenotypically and transcriptionally.

## Materials and methods

### *Candida tropicalis* isolates and growth conditions

Nine clinical isolates of *C. tropicalis* and the reference strain ATCC750 were included in the present study. The nine strains of *C. tropicalis* were selected from 68 isolates as representative strains for high or low activity of virulence factors, including adhesion, BF, and Saps. The cut-offs for classification of each virulence factor are as follows: Adhesion on polystyrene surface [low (L) < 0.004, 0.004 ≤ medium (M) < 0.015, 0.015 ≤ high (H)]; BF on polystyrene surface (L < 0.075, 0.075 ≤ M < 0.881, 0.881 ≤ H); adhesion on epithelial surface (L < 0.016, 0.016 ≤ M < 0.052, 0.052 ≤ H); BF on epithelial surface (L < 0.062, 0.062 ≤ M < 0.204, 0.204 ≤ H); and Saps and hemolysins (H < 0.41, 0.41 ≤ M < 0.61, 0.61 ≤ L < 1.00; Yu et al., 2015). The details of virulence factor activities (adhesion, BF, Saps, and hemolysins) and anatomical sites from which *C. tropicalis* were isolated are displayed in Table 1. Sample collection is coincided with the protocol of the hospital and is approved by China-Japan Friendship Hospital Ethics Committee. All strains were stored at −80°C in brain-heart infusion medium (Oxoid) and maintained in sabouraud dextrose agar media (SDA, Oxoid) at 25°C during the study. Yeast cells were inoculated in sabouraud dextrose broth (SDB, Oxoid) and incubated for 18 h at 120 rpm at 37°C. After incubation, the cells were harvested by centrifugation at 5000 × g for 5 min and washed twice with phosphate buffered saline (PBS). The washed yeast cells were used in the subsequent assays.

**Table 1:** **Virulence attributes and anatomic source of *C. tropicalis***.

**Isolates**	**PrZ_48h**	**HZ_72h**	**PMP**		**TCC-SUP**		**PZ_72h**	**Filamentation ability**	**Percentage of cell damage**	**Anatomic source**
			**LA**	**BF_CV**	**Ad_XTT**	**BF_XTT**				
CYCT01	0.504 (M)	0.474 (M)	0.014 (M)	0.001 (L)	0.019 (M)	0.018 (L)	0.564 (M)	M	0.1448 (M)	feces
FXCT01	0.443 (M)	0.461 (M)	0.003 (L)	0.052 (L)	0.046 (M)	0.187 (M)	0.567 (M)	L	0.3404 (H)	blood
ZRCT08	0.452 (M)	0.535 (M)	0.033 (H)	0.187 (M)	0.112 (H)	0.177 (M)	0.512 (M)	L	0.2178 (M)	sputum
ZRCT28	0.394 (H)	0.496 (M)	0.005 (M)	0.001 (L)	0.065 (H)	0.031 (L)	0.490 (M)	H	0.1745 (M)	urea
ZRCT32	0.465 (M)	0.442 (M)	0.018 (H)	2.954 (H)	0.024 (M)	0.200 (M)	0.474 (M)	H	0.0328 (L)	feces
ZRCT45	0.596 (M)	0.515 (M)	0.019 (H)	1.576 (H)	0.186 (H)	0.248 (H)	0.512 (M)	N	0.2197 (M)	sputum
ZRCT52	0.461 (M)	0.444 (M)	0.009 (M)	0.023 (L)	0.006 (L)	0.171 (M)	0.505 (M)	H	0.0234 (L)	sputum
ZRCT63	0.585 (M)	0.518 (M)	0.014 (M)	1.404 (H)	0.041 (M)	0.552 (H)	0.519 (M)	L	0.0543 (L)	sputum
ZRCT64	0.626 (L)	0.514 (M)	0.003 (L)	0.351 (M)	0.012 (L)	0.045 (L)	0.657 (L)	M	0.0604 (L)	sputum
ATCC750	0.413 (M)	0.557 (M)	0.033 (H)	0.171 (M)	0.022 (M)	0.024 (L)	0.508 (M)	M	0.1939 (M)	bronchus

“*H” indicates high activity; “M” indicates medium activity; and “L” indicates low activity*.

*PrZ_48 h = colony diameter/total diameter of the colony plus precipitation halo; which indicates the activity of secreted aspartyl proteases*.

*HZ_72 h = colony diameter/total diameter of the colony plus hemolysis halo; which indicates the activity of hemolysins*.

*PZ_72 h = colony diameter/total diameter of the colony plus precipitation halo; which indicates the activity of esterases*.

*PMP, polystyrene flat-bottomed microtitre plates; TCC-SUP, human urinary bladder epithelial cell line. CV, crystal violet; XTT, 2, 3-bis (2-methoxy-4-nitro-5-sulfo-phenyl)-2H-tetrazolium-5-carboxanilide; LA, late adhesion on PMP surface; BF_CV, BF on PMP surface determined by CV assay; Ad_XTT, adhesion on TCC-SUP surface quentified by XTT assay; BF_XTT, BF on TCC-SUP surface quantified by XTT assay*.

*The cut-offs for classification are as follows: PrZ_48 h, HZ_72 h, and PZ_72 h (H < 0.41, 0.41 ≤ M < 0.61, 0.61 ≤ L < 1.00); LA (L < 0.004, 0.004 ≤ M < 0.015, 0.015 ≤ H); BF_CV (L < 0.075, 0.075 ≤ M < 0.881, 0.881 ≤ H); Ad_XTT (L < 0.016, 0.016 ≤ M < 0.052, 0.052 ≤ H); and BF_XTT (L < 0.062, 0.062 ≤ M < 0.204, 0.204 ≤ H)*.

### Phenotypical analysis of virulence factors

#### Esterase and lipase activity

Esterase and lipase activity were determined using Tween 80 opacity test medium (Galan-Ladero et al., 2010) and tributyrin agar medium (Buzzini and Martini, 2002), respectively. The Tween 80 opacity medium was prepared with 5.0 g peptone, 2.5 g NaCl, 0.05 g CaCl_2_, and 7.5 g agar in 497.5 ml distilled water, adjusted to pH 6.8 and autoclaved. When the medium cooled (50°C), 2.5 ml of Tween 80 was added. The tributyrin agar medium was prepared with 2.5 g peptone, 1.5 g yeast extract and 7.5 g agar in 495 ml distilled water, adjusted to pH 6.0 and autoclaved. When the medium cooled (50°C), 5.0 ml tributyrin was added. Five microliters of yeast suspension (10^8^ CFU ml^−1^) was dropped onto each test medium. The plates were incubated for 24, 48, and 72 h at 37°C. The activity of esterase and lipase was expressed according to the PZ index (colony diameter/total diameter of the colony plus the precipitation halo). The levels of hydrolytic enzymes activity were established according to the following range of PZ index: PZ < 0.41, high; 0.41 ≤ PZ < 0.61, medium; 0.61 ≤ PZ < 1.00, low; PZ = 1.00, none (Yu et al., 2015).

#### Filamentation ability

The filamentation ability of *C. tropicalis* was determined microscopically according to Galan-Ladero et al., with some modifications (Galan-Ladero et al., 2013). Briefly, *C. tropicalis* colonies from SDA plates were incubated into yeast extract-peptone-dextrose (YEPD) broth (Lackey et al., 2013) and incubated at 120 rpm at 30°C overnight. These cultures were harvested by centrifugation at 5000 × g for 5 min, washed twice with PBS and used to inoculate in non-inducing medium (YEPD, 30°C) and inducing medium (0.67% yeast nitrogen base + 0.75% glucose + 50% fetal bovine serum, 37°C; Lackey et al., 2013) at an initial optical density of 1.0 at 490 nm, and the cultures were shaken at 200 rpm for 2 h. Finally, one drop of inoculum was observed at 40x magnification to evaluate the hyphal morphology under an inverted light microscopy (Nikon Eclipse Ti-S). All experiments were repeated at least three times for each strain.

### Analysis of virulence genes expression

#### Epithelial cells

Human urinary bladder epithelial cell line (TCC-SUP) was used in this study. Cells were cultured in Minimum Essential Medium (Gibco) containing 10% fetal bovine serum (Gibco) supplemented with MEM Non-Essential Amino Acids Solution (Gibco) and Sodium Pyruvate (Gibco) in cell culture flasks at 37°C with 5% CO_2_. Cells were washed off using 0.25% trypsin-EDTA solution (Gibco). The cellular density was adjusted to 1 × 10^5^ cells ml^−1^ in fresh Minimum Essential Medium using a Neubauer chamber. Two milliliter of the suspension was added to a 12-well-plate and incubated for 24 h. Prior to inoculate with yeast suspension, the wells were washed twice with PBS.

#### RNA extraction

The washed yeast cells were suspended in minimum essential medium (Gibco), and the density of suspension was adjusted to 1 × 10^7^ cells ml^−1^. Four milliliters of yeast suspension of each *C. tropicalis* was then added to corresponding wells in the absence of TCC-SUP or containing a confluent layer of TCC-SUP, respectively. After incubation at 37°C with 5% CO_2_ for 24 h, the culture medium was analyzed for *C. tropicalis*-induced damage using a lactate dehydrogenase (LDH) assay. The wells were then rinsed twice with PBS to remove non-adherent *Candida* cells. *C. tropicalis* cells attached to polystyrene flat-bottomed microtiter plates (PMP, Corning) and TCC-SUP cells were scrapped into 600 μl Buffer RLT (Qiagen). Before, RNA extraction, glass beads (0.5 mm diameter, ~500 μl) were added and the tubes were homogenized twice for 30 s, using a TissueLyser LT (Qiagen). After disruption of the yeast cells, the RNeasy Mini kit (Qiagen) was used to extract total RNA, according to the manufacturer's recommended protocol. Potential DNA contamination was removed by RNase-free DNase I (Qiagen) treatment. The purified RNA from all samples was confirmed as DNA-free by real time PCR using *C. tropicalis ACT1* (actin) gene primers. Additionally, RNA was also extracted, following the same approach, from *C. tropicalis* planktonic cells (the washed yeast cells). To synthesize complementary DNA (cDNA), the GoScript™ Reverse Transcription System (Promega) was used according to the manufacturer's instructions.

#### Primer design

The primers for *SAPT3* and *ACT1* used for real time PCR were as described by Silva et al. (2011a). The other primers (for *ALST1-3, LIP1, LIP4, SAPT1-2*, and *SAPT4*) were designed using the primer premier 5.0 software. Primers were checked for specificity using Primer-BLAST (http://www.ncbi.nlm.nih.gov/tools/primer-blast). In addition, to verify the specificity of the newly designed primer pairs for their corresponding target genes, PCR products were amplified from ATCC750 genomic DNA. The primer sequences are listed in Table 2.

**Table 2:** ***Candida tropicalis* primers used for reverse transcriptase PCR analysis of virulence and control gene expression**.

**Sequence (5′–3′)**	**Primer**	**Target**	**PCR product size (bp)**	**Source**
GGGCTCTGGTCGTGATGT	Forward	*ALS1*	164	This study
GTGAGGGAATGAGTCTTG	Reverse			
ACTCGTGCCTATACCTAC	Forward	*ALS2*	80	This study
TTGTTGCCGTAATGGTGG	Reverse			
AGGTGCTGTAGTTGTTCTT	Forward	*ALS3*	81	This study
AGCAGTCGGGTTGAAAGG	Reverse			
TGGGCAGCACCAATCAAAT	Forward	*LIP1*	194	This study
GGGTAGACAATCGGGACA	Reverse			
TTGACTGTGCTCCTTCCT	Forward	*LIP4*	138	This study
GCTTTGGACCTTCGTAAT	Reverse			
TATGACAATGTGCCAGTT	Forward	*SAP1*	150	This study
TAAAGCAGTCAAAGTCCC	Reverse			
GCTGGTTTCTGTGCTTTG	Forward	*SAP2*	82	This study
CCACGTAGGCATGTCTTA	Reverse			
ACTTGGATTTCCAGCGAAGA	Forward	*SAP3*	165	Silva et al., 2011a
AGCCCTTCCAATGCCTAAAT	Reverse			
CTTCACCTCCTGGTTTCATTTC	Forward	*SAP4*	217	This study
TCAACTACCCATAAATCAGAGG	Reverse			
GACCGAAGCTCCAATGAATC	Forward	*ACT1*	181	Silva et al., 2011a
AATTGGGACAACGTGGGTAA	Reverse			

#### Real time PCR

Real time PCR was used to determine the relative levels the mRNA transcripts of virulence genes (*ALST1-3, LIP1, LIP4*, and *SAPT1-4*) in the RNA samples, with *ACT1* used as a reference housekeeping gene. Gene expression was assessed by the ΔC_T_ method, using the control gene (*ACT1*) to normalize the data. Each reaction was performed in triplicate and mean values of relative expression were analyzed for each gene.

### Epithelial cell damage assay

The release of LDH by epithelial cells into the culture medium was used as a measure of cell damage. The LDH concentration in the medium was measured at 2, 6, 12, 24, and 48 h using the CytoTox-ONE™ kit (Promega), in accordance with the manufacturer's instructions. Two controls for LDH activity were prepared: (I) epithelial cells grown in the absence of *C. tropicalis*; and (II) yeast cells as the sole culture. The LDH activity was analyzed according to the method of Negri et al. (2011). In addition, colonization and morphological characteristics of *C. tropicalis* on TCC-SUP surface were confirmed by microscopic observation (Nikon Eclipse Ti-S, 40x magnification) of cells stained with periodic acid-Schiff stain (PAS, Sigma). All experiments were performed in triplicate.

### Statistical analysis

Analyses were performed using the SPSS 22.0 program. The Wilcoxon signed-rank test was used for comparison differences between two groups. The Friedman *M* test was used to determine statistically significant differences among groups. Spearman's rank correlation was used for correlation analysis. In addition, “heatmap” in R was used to illustrate the differential expression of virulence genes graphically. In brief, the heat map was generated by a log transformation of the RT-PCR data, presented as values of relative expression. Clustering was performed by average linkage and Euclidean distances, used as a distances measure for both dimensions. *P* < 0.05 was considered to be statistical significance.

## Results

### Phenotypical analysis of virulence factors *in vitro*

**Figure 1:**
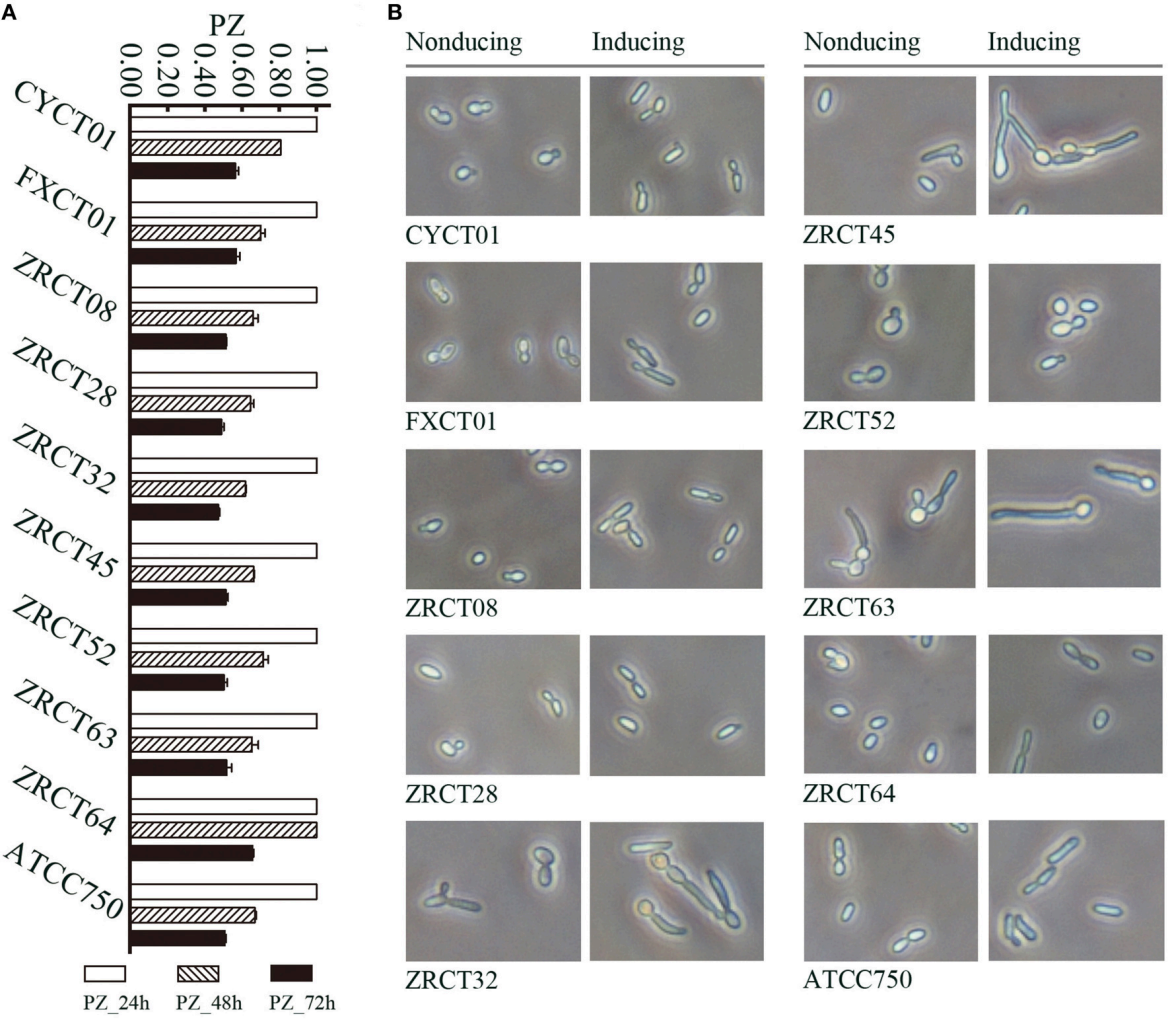
**Phenotypical analysis of virulence factors for *C. tropicalis in vitro*. (A)** Esterase activity of *C. tropicalis* at differnet time points (24, 48, and 72 h). **(B)** Filamentation ability of *C. tropicalis* in non-inducing and inducing medium.

Analysis of the adhesion, BF and hydrolytic enzymes (including Saps, phospholipases, and hemolysins) for *C. tropicalis* indicated that all isolates could produce adhesion, BF, Saps, and hemolysins with strain-dependent features, while no phospholipases were detected (Yu et al., 2015). *C. tropicalis* did not display esterase activity at 24 h (Figure 1A). However, all 10 strains showed low activity of esterase, except for strain ZRCT64, at 48 h. At 72 h, strain ZRCT64 showed low esterase activity and the other isolates exhibited medium esterase activity (Figure 1A and Table 1). It indicated that the esterase activity would be strengthened with the extension time. However, lipase activity was not detected at 24, 48, 72 h or even longer (96 and 120 h) in any of the 10 *C. tropicalis* isolates (data not shown).

The filamentation ability of *C. tropicalis* was evaluated under inducing condition *in vitro*. In non-inducing medium, strains CYCT01, FXCT01, ZRCT08, ZRCT28, ZRCT52, ZRCT64, and ATCC750 were mainly present as yeast cells, but for ZRCT32, ZRCT45, and ZRCT63, some pseudohyphae or hyphae appeared after 2 h of incubation (Figure 1B). In inducing medium, ZRCT52 still presented as yeast cells; however, the other isolates appeared as elongated yeast cells and filamentous cells to varying degrees (Figure 1B and Supplementary Material). Concerning to the form of *C. tropicalis* in non-inducing medium and inducing medium, the filamentation ability of ZRCT32, ZRCT45, and ZRCT63 was strong; FXCT01 and ATCC750 showed medium filamentation ability; CYCT01, ZRCT08, ZRCT28, and ZRCT64 had low filamentation ability; and ZRCT52 displayed no filamentation ability (Table 1). Table 1 shows the BF on PMP and TCC-SUP surfaces of the 10 strains. Interestingly, *C. tropicalis* strains with strong filamentation abilities also displayed high BF on PMP (ZRCT32, ZRCT45, and ZRCT63) and TCC-SUP (ZRCT45 and ZRCT63). Significant correlation was found between the level of filamentation ability and BF on PMP (*r*_*s*_ = 0.737, *P* = 0.015), and TCC-SUP (*r*_*s*_ = 0.674, *P* = 0.033).

### The expression profile of virulence genes in *C. tropicalis*

**Figure 2:**
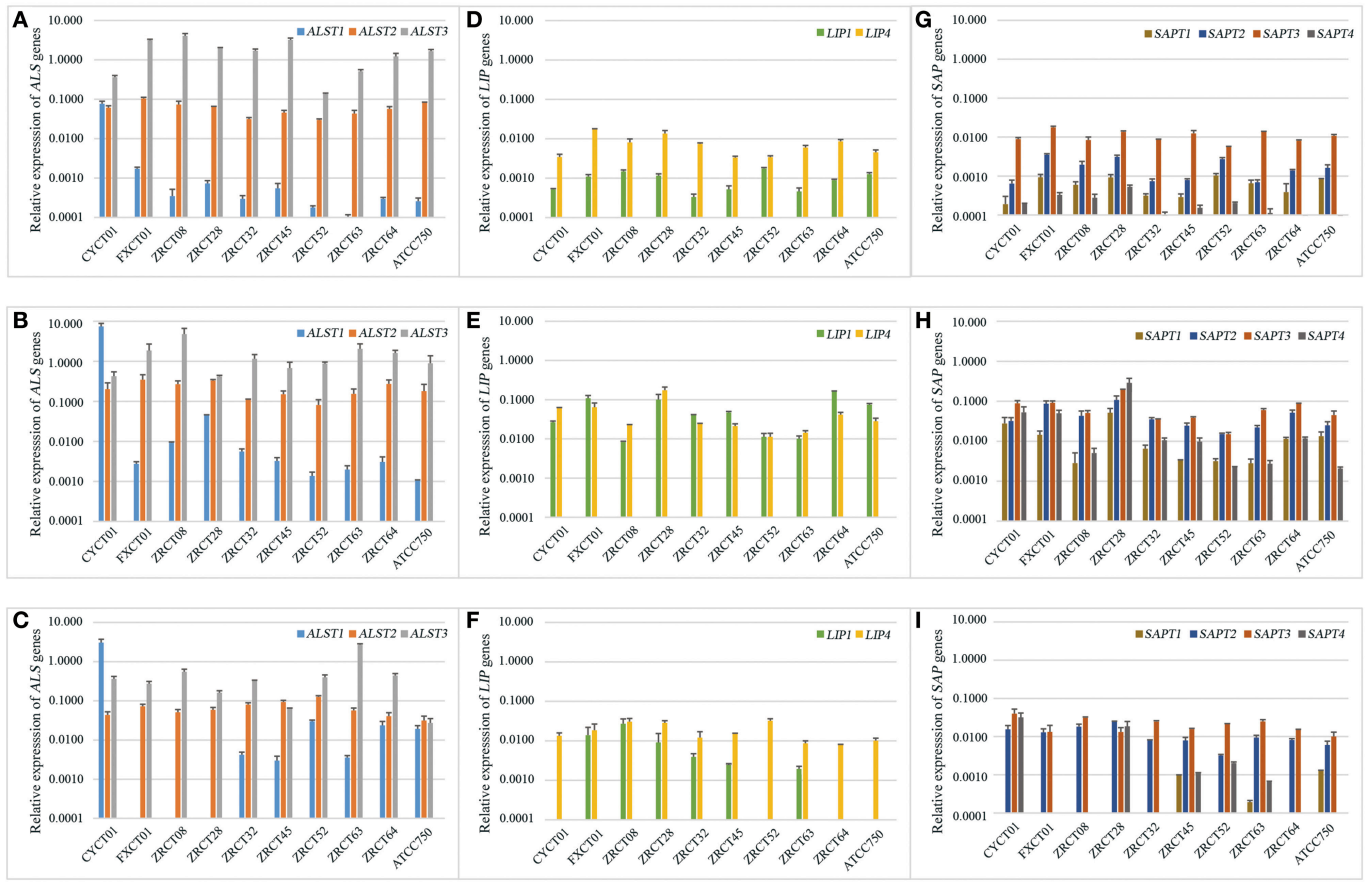
**The expression profile of virulence genes including *ALS1-3*, *LIP1*, *LIP4*, and *SAP1-4* in three different conditions. (A)** The expression of *ALST1-3* genes in planktonic; **(B)** The expression of *ALST1-3* genes on PMP surface; **(C)** The expression of *ALST1-3* genes on TCC-SUP surface; **(D)** The expression of *LIP1* and *LIP4* genes in planktonic; **(E)** The expression of *LIP1* and *LIP4* genes on PMP surface; **(F)** The expression of *LIP1* and *LIP4* genes on TCC-SUP surface; **(G)** The expression of *SAPT1-4* genes in planktonic; **(H)** The expression of *SAPT1-4* genes on PMP surface; **(I)** The expression of *SAPT1-4* genes on TCC-SUP surface.

Reverse transcription PCR (RT-PCR) analysis revealed a wide range of expression profiles of virulence genes in the three conditions examined (Figure 2). For the *ALS* genes, *ALST3* showed the highest expression in the three conditions for all *C. tropicalis* strains except CYCT01, ZRCT45, and ATCC750 (Figures 2A–C). Strain CYCT01 showed the highest expression level of *ALST1* on PMP and TCC-SUP surfaces (7.46 and 2.93, respectively, Figures 2B,C). Meanwhile strains ZRCT45 and ZRCT750 exhibited the highest expression level of *ALST2* on the TCC-SUP surface. Comparing the expressions of the *ALS* genes in yeast grown on three different status, there was an increase in the expression of *ALST1-2* on the PMP surface compared with the planktonic and TCC-SUP surface (*P* < 0.05). However, the expression of *ALST3* was similar between the planktonic and on the PMP surface for most *C. tropicalis* strains (*P* = 0.508; Figures 2A–C).

For the *LIP* genes, all *C. tropicalis* strains exhibited higher expression of *LIP4* compared with *LIP1* in planktonic (*P* = 0.005) and on TCC-SUP surface (*P* = 0.005), while the expressions of *LIP1* and *LIP4* on the PMP surface were similar (*P* = 0.508; Figures 2D–F). There was an apparent increase in the expression of *LIP* genes on PMP surface compared with that in planktonic and on TCC-SUP surface for all *C. tropicalis* strains except ZRCT08 and ZRCT52. Strain ZRCT08 exhibited the highest expression of *LIP1*, and strain ZRCT52 displayed the highest expression of *LIP4* on the TCC-SUP surface (Figures 2D–F).

For the *SAP* genes, the expression of *SAPT3* was the highest for all *C. tropicalis* strain, except for ZRCT28 under the three different conditions (Figures 2G–I). Isolate ZRCT28 showed high activity of Saps *in vitro*, of which the expressions of *SAPT4* and *SAPT1* were the highest on PMP and TCC-SUP surfaces, respectively, (Figures 2H,I). The expressions of *SAP* genes on the PMP surface were upregulated compared with those in planktonic and on TCC-SUP surface for all *C. tropicalis* strains (*P* < 0.05; Figures 2G–I).

In general, virulence genes transcripts were all detected in cells grown in planktonic and on polystyrene surface, whereas some virulence genes were rarely detected on the TCC-SUP surface (Figure 2). *ALST3* and *SAPT3* were the highest expressed genes for most *C. tropicalis*. In addition, all detected genes, except for *ALST3*, exhibited their highest expression level on the PMP surface compared with that in planktonic cell and on TCC-SUP surface (Figure 2).

### Correlation between virulence genes expression and its phenotype

The above analysis showed that *C. tropicalis* exhibited diverse and strain-dependent differences with respect to phenotype and expression of virulence factors. The correlation between transcription and the phenotype of virulence factors was investigated. For Saps, a significant correlation existed between the expression of *SAPT1* and *SAPT2* in planktonic and the activity of Saps (*r*_*s*_ values were 0.636 and 0.685, respectively, Table 3). For adhesion on the PMP surface, a negative correlation was observed between the expressions of *LIP1, LIP4*, and *SAPT1* on the PMP surface and adhesion (LA). The *r*_*s*_ values were −0.878, −0.673, and −0.733 and the *P*-values were 0.008, 0.033, and 0.016, respectively, (Table 3). For adhesion on the TCC-SUP surface, a negative correlation was observed between the expression of *ALST1* on the TCC-SUP surface and adhesion (XTT; *r*_*s*_ = −0.877, *P* = 0.001; Table 3). And a positive correlation was noted between the expression of *LIP1* on the TCC-SUP surface and adhesion (XTT; *r*_*s*_ = 0.807, *P* = 0.005; Table 3). However, the activity of hemolysins and BF did not display significant association with the expression of a single virulence gene.

**Table 3:** **The correlation between virulence genes expression and its phenotype in *C. tropicalis***.

**Relative expression**	**Planktonic**		**PMP**		**TCC-SUP**	
	**PrZ_48 h**	**HZ_72 h**	**LA**	**BF_CV**	**Ad_XTT**	**BF_XTT**
*ALST1*	−0.152	−0.200	0.079	−0.188	−0.877^*^	−0.436
*ALST2*	−0.564	0.418	−0.539	−0.442	0.212	0.564
*ALST3*	−0.261	0.285	0.333	0.479	−0.236	0.261
*LIP1*	−0.564	0.224	−0.782^*^	−0.127	0.807^*^	0.431
*LIP4*	−0.382	−0.091	−0.673^*^	−0.503	0.273	−0.055
*SAPT1*	−0.636^*^	−0.091	−0.733^*^	−0.612	0.231	0.216
*SAPT2*	−0.685^*^	−0.091	−0.564	−0.273	0.479	−0.155
*SAPT3*	−0.261	0.164	−0.491	−0.467	−0.042	0.212
*SAPT4*	−0.479	−0.285	−0.467	−0.358	−0.071	−0.278

*PrZ_48 h and HZ_72 h indicate the activity of secreted aspartyl proteases and hemolysins, respectively*.

*PMP, polystyrene flat-bottomed microtitre plates; TCC-SUP, human urinary bladder epithelial cell line. CV, crystal violet; XTT, 2, 3-bis (2-methoxy-4-nitro-5-sulfo-phenyl)-2H-tetrazolium-5-carboxanilide; LA, late adhesion on PMP surface; BF_CV, BF on PMP surface determined by CV assay; Ad_XTT, adhesion on TCC-SUP surface quentified by XTT assay; BF_XTT, BF on TCC-SUP surface quantified by XTT assay*.

* *Correlation is significant at the 0.05 level (2-tailed)*.

**Figure 3:**
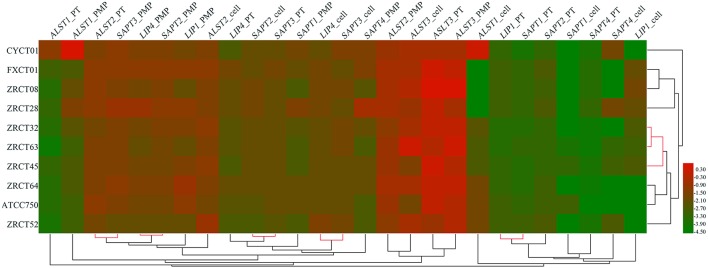
**Clustering analysis of the virulence genes expression for *C. tropicalis* in heat map**. The heat map was generated by a log transformation of the reverse transcriptase PCR data presented as values of relative expression, and the clustering was performed by average linkage and Euclidean distances used as a distances measure for both dimensions. Red indicates high intensity of gene expression; and green indicates low intensity.

The expression of nine virulence genes among the 10 *C. tropicalis* isolates was analyzed using a heat map (Figure 3). We observed that the *LIP* and *SAP* genes in same status were closely related, such as the expression of *LIP1* and *SAPT1* in planktonic; *LIP4* and *SAPT2* in cells grown on the PMP surface; and *LIP4* and *SAPT3* in cells grown on the TCC-SUP surface (Figure 3). Moreover, some virulence genes detected in different conditions displayed remarkable relationships. For example, the expression of *ALST2* in planktonic and *SAPT3* in cells grown on the PMP surface; and *SAPT2* in cells grown on the TCC-SUP surface and *SAPT3* in planktonic (Figure 3) were all closely related with each other. Interestingly, hierarchical clustering analysis of rows (strains) illustrated that the strains with a high BF (ZRCT32, ZRCT45, and ZRCT63) on the PMP surface were closely related, while the isolates (CYCT01, FXCT01, ZRCT28, and ZRCT52) with a low BF on the PMP surface exhibited a dispersed relation (Figure 3).

### Analysis of the association of virulence factors with cell damage induced by *C. tropicalis* phenotypically and transcriptionally

**Figure 4:**
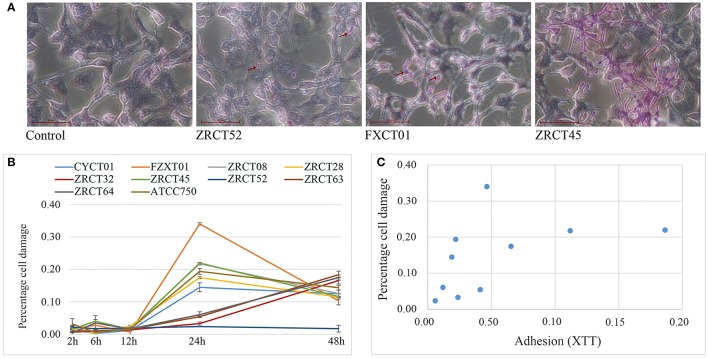
**The epithelial cell (TCC-SUP) infected with *C. tropicalis*. (A)** Light micrographs of *C. tropicalis* infecting TCC-SUP after 12 h incubation (stained with periodic acid-Schiff stain). The red arrows points to the colonized *C. tropicalis*. **(B)** Percentages of cell damage induced by *C. tropicalis* with time. **(C)** The relationship between adhesion on TCC-SUP and cell damage.

The colonization and filamentation of *C. tropicalis* on the TCC-SUP surface was evaluated after incubation for 12 h using light microscopy (Figure 4A). All *C. tropicalis* strains could colonize the TCC-SUP surface, but the extent of colonization was strain-dependent. Strains ZRCT45 and ZRCT63 showed high colonization; strains FXCT01, ZRCT08, ZRCT32, and ATCC750 displayed medium colonization; while CYCT01, ZRCT28, ZRCT52, and ZRCT64 exhibited low colonization. The filamentation extent was also different among the *C. tropicalis* isolates on the TCC-SUP surface. Strain ZRCT52 was the only strain to colonize the TCC-SUP surface primarily in the yeast form (Figure 4A). In addition, morphological changes of epithelial cells were still detected by microscopy even in cells infected with *C. tropicalis* isolates in yeast form, particularly for strain FXCT01 (Figure 4A).

Cell damage caused by those isolates was determined at 2, 6, 12, 24, and 48 h infection using the LDH assay (Figure 4B). Co-culture of TCC-SUP cells with each isolate separately, caused slight cell damage at 2, 6, and 12 h (Figure 4B). The levels of LDH apparently increased at 24 and 48 h (*P* < 0.05; Figure 4B). Cell damage at 24 h was more pronounced than that at 48 h; therefore data of cell damage at 24 h were used in the subsequent analyses (Figure 4B). Strain FXCT01 (isolated from blood) caused the most serious cell damage (34.04%) at 24 h (Figure 4B). Furthermore, ZRCT52 with low adhesion on the TCC-SUP surface and no filamentation ability, caused the least cell damage (2.34%; Figure 4B). Strains ZRCT32, ZRCT63, and ZRCT64 also caused a relatively low percentage of cell damage at 24 h; however, the cell damage increased at 48 h (Figure 4B). The remaining strains (CYCT01, ZRCT08, ZRCT28, ZRCT45, and ATCC750) induced moderate cell damage. Statistical analysis showed that the damage caused by *C. tropicalis* significantly correlated with adhesion on the TCC-SUP surface (*r*_*s*_ = 0.697, *P* = 0.025; Figure 4C).

Three genes associated adhesion (*ALST1, ALST2*, and *ALST3*) showed a significant correlation with cell damage (24 h). As the expression of *ALST1* on TCC-SUP surface decreased, cell damage became more severe (*r*_*s*_ = −0.632, *P* = 0.050). While positive correlation existed between the cell damage (24 h) and the expression of *ALST2* and *ALST3* in planktonic (*r*_*s*_ was 0.806 and 0.770, respectively). However, there was no significant correlation between expression of other virulence genes (*LIP1, LIP4*, and *SAPT1-4*) and cell damage.

## Discussion

*C. tropicalis*, an emerging opportunistic pathogen, mainly cause candidosis in immunocompromised patients (Guinea, 2014). The pathogenesis of *Candida* is promoted by a number of factors, including adhesion, BF, filamentation, and secreted hydrolytic enzymes (Silva et al., 2011b). However, compared with *C. albicans*, relatively little is known about the virulence of *C. tropicalis*. Phylogenetically, *C. tropicalis* is close to *C. albicans* (Butler et al., 2009). Both of them belong in a single *Candida* clade characterized by the unique translation of CUG codons, both are diploid species and have the similar Major Repeat Sequences (MRSs; Butler et al., 2009). Thus, studies on *C. albicans* will give some hints to the pathogenicity of *C. tropicalis*. In the present study, the expression profiles of virulence genes, including *ALS, LIP*, and *SAP* genes, were firstly analyzed in cells growing in planktonic, on polystyrene and TCC-SUP surfaces. In addition, we attempted to specify the role of virulence factors in the cell damage induced by *C. tropicalis* at the level of transcription and phenotype.

The secretion of hydrolytic enzymes is reported to play an important role in the pathogenicity *of Candida* (Silva et al., 2011b). While Saps of *C. tropicalis* have been well characterized, secreted hydrolytic lipases and esterase have been neglected, and might play an important role in the pathogenicity of candidosis (Schaller et al., 2005; Gacser et al., 2007a,b). In this study, the lipase and esterase activities of *C. tropicalis* were determined using the plate method (Buzzini and Martini, 2002; Galan-Ladero et al., 2010). All *C. tropicalis* isolates used in this study displayed esterase activity and the esterase activity increased in a time dependent manner. This result agreed with previous reports (Slifkin, 2000; Galan-Ladero et al., 2010). However, no lipase activity was detected in the *C. tropicalis* strains used in this study. This might be because the plate assay is relatively insensitive and could not detect the activity in low-lipase-producing strains (Kouker and Jaeger, 1987). Filamentation of *C. albicans* is required for virulence and is important for several virulence-related processes, including invasion of epithelial cell layers, and BF (Kumamoto and Vinces, 2005; Lackey et al., 2013). We evaluated the filamentation ability of *C. tropicalis* under inducing conditions (serum and 37°C). All tested *C. tropicalis* isolates, except for ZRCT52, displayed filamentous growth in inducing medium. Filamentation of *C. tropicalis, C*. *parasilosis* and *C*. *guilliermondii* is inhibited in medium containing rich nutrients, such as YEPD, which suggests that the nutrient-sensing pathway may play a conserved role in repressing the yeast-filament transition for multiple non-*albicans Candida* species (Lackey et al., 2013). In addition, that was a significant correlation between filamentation ability and BF in *C. tropicalis*, which is supported by previous reports (Galan-Ladero et al., 2013; Moralez et al., 2014). Furthermore, Sherry *et al* showed that individual genes were less informative than multiple genes combined with adhesion, hydrolytic enzymes, filamentation and resistance, when trying to classify isolates as low BF or high BF strains of *C. albicans* (Sherry et al., 2014). Our results also showed that *C. tropicalis* isolates with high BF abilities were clustered together according to their expressions of the nine virulence genes, but not according to the expression of a single virulence gene.

The adhesions, lipases and Saps are encoded by *ALS, LIP* and *SAP* genes (*SAPT1-4*), respectively, (Silva et al., 2011b). There are 16 *ALS* genes and five *LIP* genes in the *C. tropicalis* (MYA 3404) genome sequence (Butler et al., 2009). However, we only found three *ALS* genes (*ALST1*: AF201686.1; *ALST2*: AF211865.1; and *ALST3*: AF211866.1), two *LIP* genes (*LIP1*: XM_002549845.1 and *LIP4*: XM_002548709.1) and four *SAP* genes (*SAPT1*: X61438.1; *SAPT2*: AF115320.1; *SAPT3*: AF115321.1; and *SAPT4*: AF115322.1) in GenBank. Therefore, we explored the expression profiles of *ALS1-3, LIP1, LIP4*, and *SAPT1-4* genes in three different conditions. RT-PCR analysis revealed that the expressions of *ALST3* and *SAPT3* were the highest among the *ALS* and *SAP* genes, respectively. This observation for *SAPT3* expression was similar to published results (Negri et al., 2011, 2012; Silva et al., 2011a). Additionally, Silva et al. found that there was an apparent increase in the expression of most *SAP* genes when the cells were grown on a PMP surface (Silva et al., 2011a). Our study also showed that *C. tropicalis* exhibited a higher expression level of virulence genes not only *SAP* genes, but also *ALST1-2, LIP1*, and *LIP4* genes, on the PMP surface compared with growth on epithelial and planktonic cells. Other study demonstrated that sessile *C. albicans* cells on a biotic surface secreted more aspartyl proteases than planktonic cells (Mendes et al., 2007). These results suggested that biofilm formation on polystyrene could promote the expression of other virulence genes, but the underlying mechanisms need to be further analyzed. In our previous study, a negative correlation was found between activity of Saps *in vitro* and adhesion (LA) on a PMP surface. At the same time, the expressions of *LIP1, LIP4* and *SAPT1* negatively correlated with adhesion (LA) in the present study. Another study also revealed a significant negative correlation between *SAPT3* and the biomass data in *C. albicans* (Sherry et al., 2014). Our study revealed that the activity of Saps in a medium containing bovine serum albumin (BSA) as the sole source of nitrogen was associated with the expression of *SAPT1* and *SAPT2* in planktonic, which was in accordance with Zaugg's results that the gene products (Sap1p) was the dominant product in BSA medium (Zaugg et al., 2001). However, the majority of strains did not express *SAPT1* on the TCC-SUP surface, suggesting its limited involvement in invasion and tissue damage (Silva et al., 2011a). Additionally, no significant correlation was found between the activity of Saps or the expression of *SAP* genes and cell damage. Other investigators also showed that for *C. albicans* (Lermann and Morschhauser, 2008; Naglik et al., 2008), and *C. tropicalis* (Okawa et al., 2008; Silva et al., 2011a), Saps are not required for invasion and damage to reconstituted human oral epithelium (RHOE). Based on the present and previous results, Saps probably have a limited role in epithelial cell or tissue damage.

Our study showed that *C. tropicalis* was able to colonize and develop filamentous forms on epithelial cells (TCC-SUP) in a strain-dependent manner. The greatest cell damage was induced by *C. tropicalis* at 24 h, and the most pronounced differences were found among isolates at this time point, which is similar to results reported previously (Silva et al., 2011a). This might reflect a variation in the ability of distinct strains to continue to grow on epithelial cells and induce damage after initial invasion. The pathogenicity of *C. albicans* strains is well correlated with adherence to mucosal epithelial cells (Hela cells) and hydrophobicity (Okawa et al., 2007). Our results also showed that adhesion on the TCC-SUP surface correlated positively with cell damage. In addition, we observed significant correlation between cell damage and the expression of *ALST1-3* in *C. tropicalis*, which further confirmed the hypothesis that adhesion on epithelial cells plays an important role in subsequent cell damage. However, isolate FXCT01 from the blood is an exception: it exhibited the highest percentages of cell damage with only moderate adhesion activity. Importantly, one strain, ZRCT52, with no filamentation ability, induced the least cell damage at 24 and 48 h. This observation supported the hypothesis that filamentation plays a role in epithelial cell damage.

In conclusion, we analyzed the virulence factors of *C. tropicalis* phenotypically and transcriptionally. *C. tropicalis* displayed strain-dependent filamentation ability. Isolates with high filamentation ability also displayed high BF on polystyrene. These results suggested that filamentation plays a critical role in BF. Furthermore, *ALST3* and *SAPT3* were expressed at the highest levels in most *C. tropicalis* strains. The expression of virulence genes (*ALST1-2, LIP1, LIP4*, and *SAPT1-4*) was upregulated on the PMP surface compared with planktonic and TCC-SUP counterparts. Isolates with high BF on polystyrene formed a distinct group in the hierarchical clustering analysis of virulence genes. Moreover, adhesion to epithelial cells and the expression of *ALST2-3* encoding an adhesins, correlated positively with cell damage, which suggested that adhesion on epithelial cells plays an important role in cell damage. One strain (ZRCT52) with no filamentation ability caused the least cell damage, which might indicate that filamentation is involved in cell damage. Above all, *C. tropicalis* displayed diverse and strain-dependent differences with respect to phenotype and transcription of virulence factors. Our study will aid the further study of the invasive mechanisms of *C. tropicalis*.

## Author contributions

SY performed the experiments, analyzed the data, and drafted the manuscript. WL, XL, and JC helped in the related experiments. JL participated in the design of the experiment proposal and revision of the paper. YW conceived the study, supervised the research, and revised the manuscript.

### Conflict of interest statement

The authors declare that the research was conducted in the absence of any commercial or financial relationships that could be construed as a potential conflict of interest.
