# Supplementary figures and images for: Distinct Expression Levels of ALS, LIP, and SAP Genes in Candida tropicalis with Diverse Virulent Activities
Source: Front Microbiol. 2016 Jul 29;7:1175. doi: 10.3389/fmicb.2016.01175 (PMC4965447; doi:10.3389/fmicb.2016.01175)

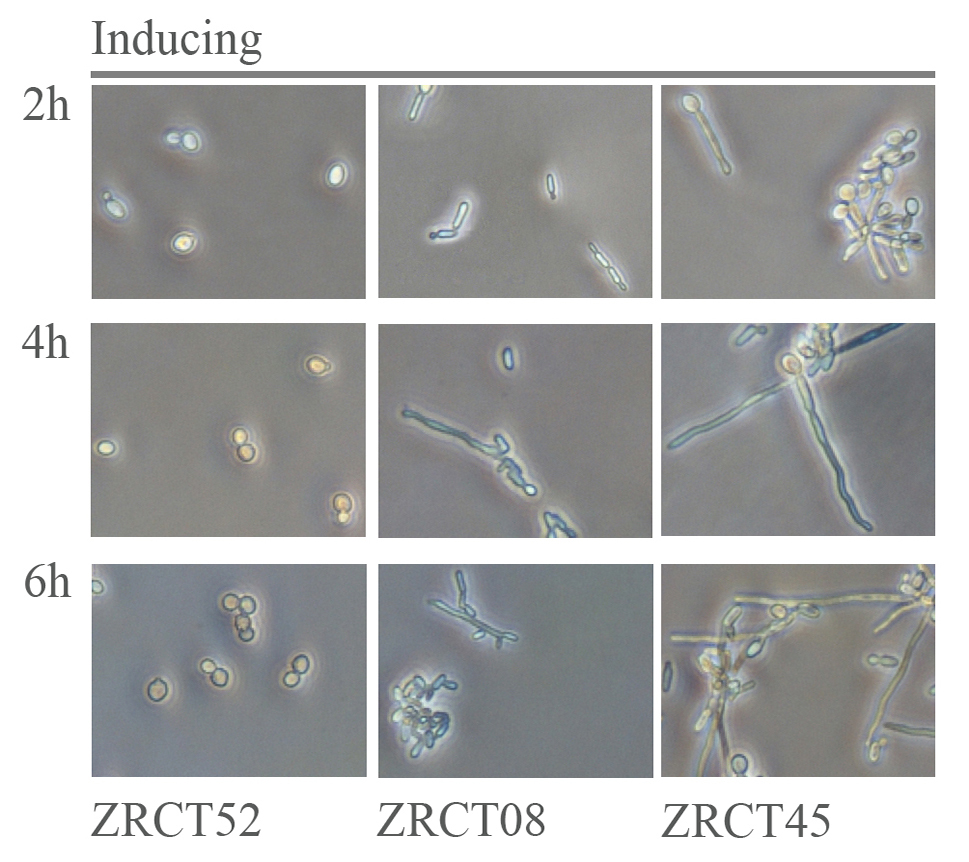

Supplement: Supplementary file 1 [file Image1.JPEG]
